# High-frequency audiometry in the diagnosis of tinnitus

**DOI:** 10.1007/s11845-023-03462-y

**Published:** 2023-07-31

**Authors:** Artur Bogacz, Anna Sinkiewicz, Paweł Burduk, Agata Kozakiewicz-Rutkowska, Agnieszka Kubala-Owieśny

**Affiliations:** Department of Otolaryngology, Phoniatrics and Audiology, Ludwik Rydygier Collegium Medicum in Bydgoszcz, Nicolaus Copernicus University in Toruń, Ujejskiego 75 Street, 85-168 Bydgoszcz, Poland

**Keywords:** Hearing loss, High-frequency audiometry, Tinnitus

## Abstract

**Background:**

Subjective tinnitus is an unpleasant perception of sound without any external acoustic stimulus. It can be manifested in the form of various phantom sounds, which most often resemble ringing, whistling, squeaking, noise, chirping, or buzzing. The sounds are heard solely by the sufferer and can occur in the middle of the head, but also in the ears—on one or both sides.

**Aim:**

The aim of the study was to evaluate the hearing capacity based on audiometric threshold measurements in the frequency range of 0.125–16 kHz in patients with tinnitus. In addition, we investigated the following questions:

- Can high-frequency audiometry be useful in the diagnosis of tinnitus?
- Does hearing loss occur in an increasingly wide frequency range with age compared to the control group?
- Can tinnitus be considered the first symptom of the onset of high-frequency hearing loss?

**Methods:**

The study included 99 patients, all of whom underwent pure-tone audiometry (PTA) and extended high-frequency audiometry (HFA) in the ranges of 0.125–8 kHz and 8–16 kHz, respectively. In each patient (excluding the control group), tinnitus was characterized in terms of its frequency and intensity.

**Results and conclusion:**

The study concluded that tinnitus may be a symptom indicating the presence of high-frequency hearing loss as hearing loss occurs in an increasingly wider frequency range with age, so HFA should be a routine audiological test in patients with tinnitus.

## Introduction

HFA is a test that measures the threshold of hearing pure tones in the range above 8 kHz. The frequencies at which the human ear can pick up sounds reach up to 20 kHz, while standard audiometric testing is performed in the range up to 8 kHz.

Currently, HFA is used in the prevention of early hearing damage caused by noise, as well as ototoxic drugs, e.g., cisplatin chemotherapy or aminoglycoside antibiotics [1–4]. HFA studies have also been conducted with chronic diseases like diabetes, hyperlipidemia, and renal failure [5–7].

In the clinical practice, this test plays a special role, because it can detect auditory damage before it appears in a conventional audiogram. If the ototraumatic factor that affects the patient is eliminated, there is theoretically a chance to stop the spread of hearing loss also to lower frequencies. Therefore, preventive hearing testing using HFA is so important. The idea of using HFA in audiological tests in patients with tinnitus was a natural consequence of observing tinnitus at frequencies above 8 kHz.

The relationship between the amount of noise and the extent of hearing loss is well described in the literature. The high-frequency nature of noise may indicate a hearing loss in the range above 8 kHz [8, 9].

The number of studies about audiometric tests in the extended high-frequency range has significantly increased, due to growing interest in this method of testing. It is important to note that the progression of the disease in the inner ear is externalized, especially in the earliest stages, by high-frequency hearing impairment.

The aim of the study was to evaluate the hearing capacity based on audiometric threshold measurements in the frequency range from 0.125 to 16 kHz. We also wanted to answer the following questions:

- Can high-frequency audiometry be useful in the diagnosis of tinnitus?
- Does hearing loss occur in an increasingly wide frequency range with age in comparison to the control group?
- Can tinnitus be treated as the first symptom of the onset of high-frequency hearing loss?

## Material and methods

### Clinical study population

**Fig. 1:**
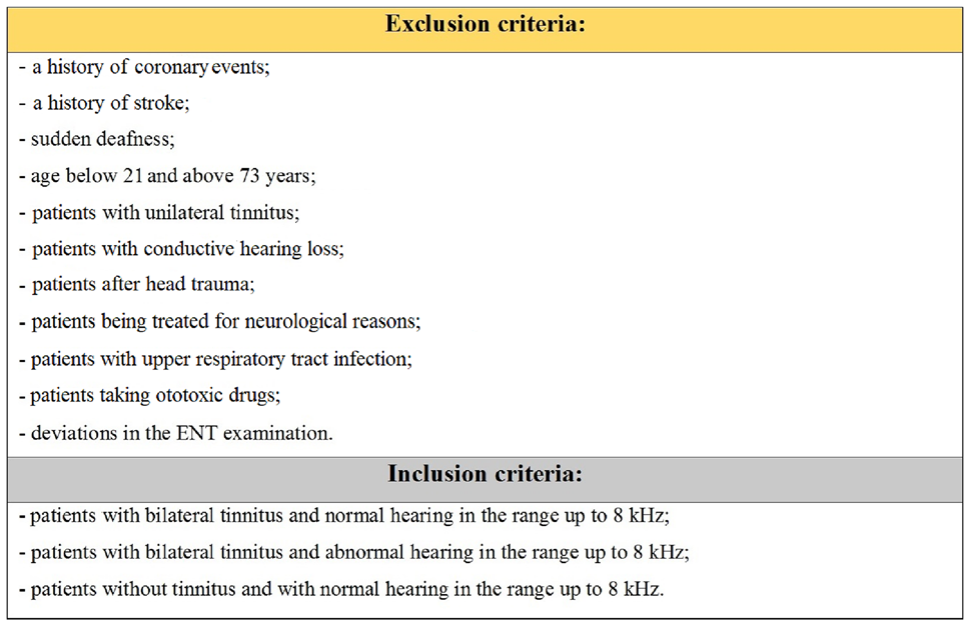
The exclusion and inclusion criteria used to select participants for the clinical trial

The clinical study population consisted of 99 patients, including 59 women and 40 men between the ages of 21 and 73. The subjects were randomly selected from 1200 patients with bilateral tinnitus qualified for the clinical trial based on the exclusion and inclusion criteria shown in Fig. 1

.

**Fig. 2:**
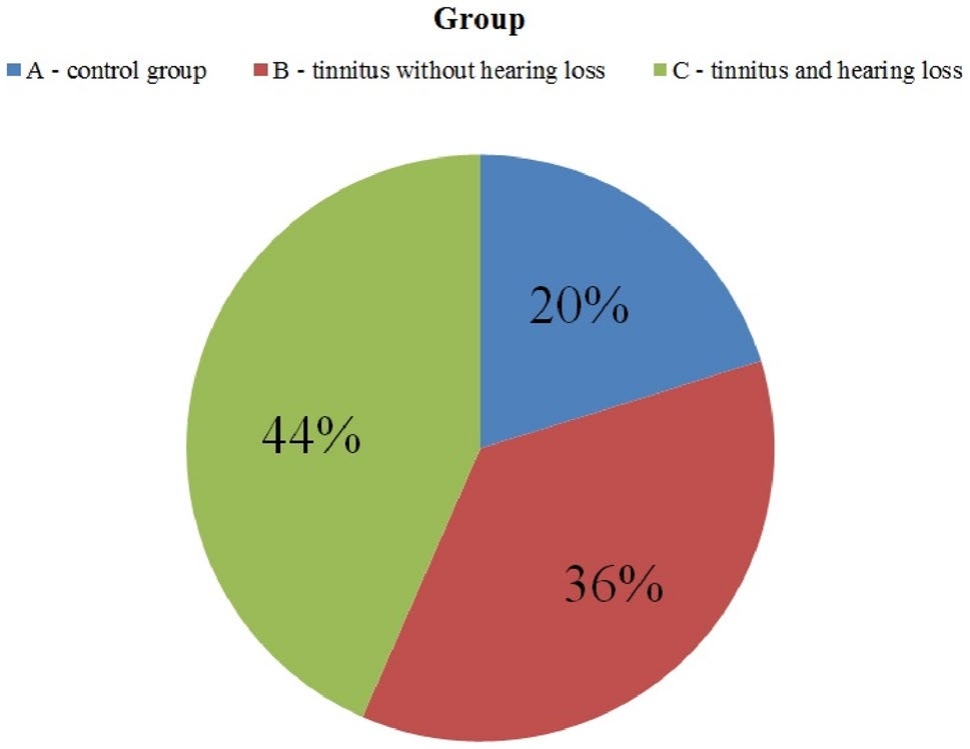
The division of the subjects into groups A, B, C

Subjects qualified for the trial were divided into three groups:

- Group A. The control group with normal hearing in PTA up to 8 kHz and without tinnitus, representing approximately 20% of the subjects qualified (20 patients with mean age of 37.9 years);
- Group B. Patients with normal hearing in PTA up to 8 kHz and with tinnitus, which accounted for approximately 36% of the subjects qualified (36 patients with mean age of 39.4 years);
- Group C. Patients with hearing loss in PTA up to 8 kHz and with tinnitus, which constituted approximately 44% of the subjects qualified (43 patients with mean age of 51.5 years) (Fig. 2).

### Clinical tests used

The hearing tests were conducted in an ear nose and throat (ENT) department during the patients’ stay in the hospital. All tests were performed by one and the same researcher.

All the patients underwent subjective and objective otolaryngological and otoneurological examination, with particular emphasis on the condition of the outer, middle, and inner ear as well as nearby anatomical areas that may affect the state of hearing or the generation of tinnitus (the nose, paranasal sinuses, and nasopharynx).

Hearing tests were performed through PTA (0.125–8 kHz) and HFA (8–16 kHz), using an Interacoustics AC40 clinical audiometer in an audiometric booth. In PTA and HFA, threshold curves were determined in the 0.125–16 kHz frequency range. The next step was to characterize tinnitus in each patient (excluding the group A) in terms of its frequency and intensity. The data collected during the study were analyzed as follows:

- hearing impairment was diagnosed based on the results of PTA and HFA;
- a sound threshold of 20 dB was considered the limit between normal hearing and hearing impairment.

### Data analysis

The significance level was set at α = 0.05 and a difference was considered statistically significant when p < 0.05. The Shapiro–Wilk test was used to check  the normality of the data distribution. Due to the nonparametric distribution of results, the Mann–Whitney *U* test was applied for comparison of two groups and the Kruskal–Wallis test was used to compare the cumulative distribution function of three groups.

## Results

Group A patients (control group) had normal results of PTA. In HFA, 90% of the results were normal and 10% were abnormal.

In group B (patients with tinnitus and normal hearing), no hearing loss was found in PTA. In HFA, 11.11% of the patients presented normal hearing, while 88.89% showed abnormal results and hearing loss.

In another group of patients with tinnitus (group C), hearing loss was found in PTA, in the 0.125–8 kHz range. Also in the extended frequency range, i.e., 8–16 kHz, 97.14% of the patients had abnormal hearing results.

The age distribution of the study groups is presented in Table 1. The mean age of persons in the control group was 37.9 years, with a median of 34 years. The distribution of results ranged between 25 and 55 years.

**Table 1:** The descriptive and statistical analysis of age distribution of the persons examined, including the division into study groups

Group	$\overline{x }$	SD	Min	*Q*_1_	Me	*Q*_3_	Max	S-W test	*P* value
A—control group	37.9	11.8	25	27	34	51	55	0.828	0.002
B—tinnitus without hearing loss	39.4	9.8	21	34	39	46	65	0.980	0.760
C—tinnitus and hearing loss	51.5	12.5	26	41	55	63	73	0.939	0.024

The mean age of patients with tinnitus and normal hearing was 39.4 years, with a median of 39. The distribution of results in this group ranged from 21 to 65 years.

Subjects with tinnitus and hearing loss belonged to the oldest age group with a mean age of 51.5 years and a median of 55 years. The age of this group ranged from 26 to 73 years.

The Shapiro–Wilk test showed statistically significant differences in age distribution between the control group (group A) versus patients with tinnitus and hearing loss (group C).

Due to the non-parametric distribution of the parameters analyzed, the Kruskal–Wallis test was used to measure between groups.

The obtained comparisons showed statistically significant differences: *P* value < 0.001. The post-hoc analysis revealed significant differences between the control group versus patients with tinnitus and hearing loss, as well as between patients with tinnitus and normal hearing versus patients with tinnitus and hearing loss. The results of post-hoc comparisons are shown in Table 2.

**Table 2:** The comparative age analysis between study groups

Dependent: age (in years)	*P* value for multiple comparisons The Kruskal–Wallis test: *H* (2, *N* = 99) = 22.247 *P* < 0.0001		
	Control group	Tinnitus without hearing loss	Tinnitus and hearing loss
A—control group			
B—tinnitus without hearing loss	1.000		
C—tinnitus and hearing loss	< 0.001	< 0.001	

**Fig. 3:**
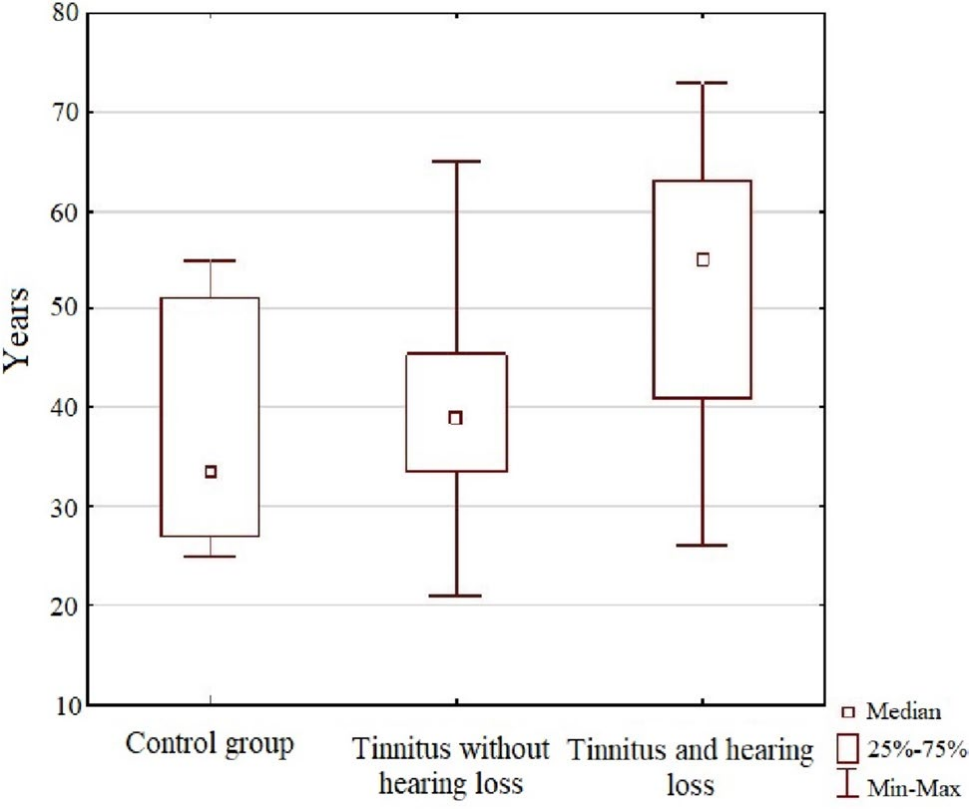
The age distribution of the patients, including the division into study groups

A graphical representation of age for the analyzed groups is shown in Fig. 3.

A comparison in terms of the descriptive and statistical analysis of tinnitus frequency and intensity parameters was also made.

The results shown in Table 3 refer to subjects who experienced tinnitus: patients with tinnitus without hearing loss and patients with both tinnitus and hearing loss.

**Table 3:** The descriptive and statistical analysis of the distribution of frequency and intensity of tinnitus in the examined patients, including the division into study groups

Group	$\overline{x }$	SD	Min	*Q*_1_	Me	*Q*_3_	Max	S-W test	*P* value
Intensity of tinnitus									
Tinnitus without hearing loss	24.17	12.85	5.0	15.0	22.5	30.0	70.0	0.870	< 0.001
Tinnitus and hearing loss	50.00	17.42	25.0	35.0	50.0	60.0	90.0	0.945	< 0.001
Frequency of tinnitus									
Tinnitus without hearing loss	3.95	3.98	0.1	0.9	3.0	6.0	16.0	0.847	0.001
Tinnitus and hearing loss	2.94	2.42	0.3	1.0	2.0	4.0	8.0	0.837	0.040

The obtained results regarding the intensity of tinnitus showed a significant difference in the mean and median values received for the compared groups. Patients with tinnitus alone achieved a mean of 24.17 dB and a median of 22.5 dB. In the case of patients with tinnitus and hearing loss, the mean and median values were twice higher, rising to 50.00 dB.

The analysis of tinnitus frequency parameters did not indicate such spectacular differences. The mean values for the group without hearing loss were slightly higher at 3.95 kHz, with a median at 3 kHz. The results obtained for patients with tinnitus and hearing loss reached a mean of 2.94 kHz and a median of 2 kHz.

In each of the analyzed cases, the values did not have a normal distribution.

Due to the nonparametric distribution of results, comparisons were performed using the Mann–Whitney *U* test.

The comparative analyses of frequency and intensity of tinnitus showed statistically significant differences only in the second case. The intensity of tinnitus was higher in patients with tinnitus and hearing loss compared to patients with tinnitus alone.

**Fig. 4:**
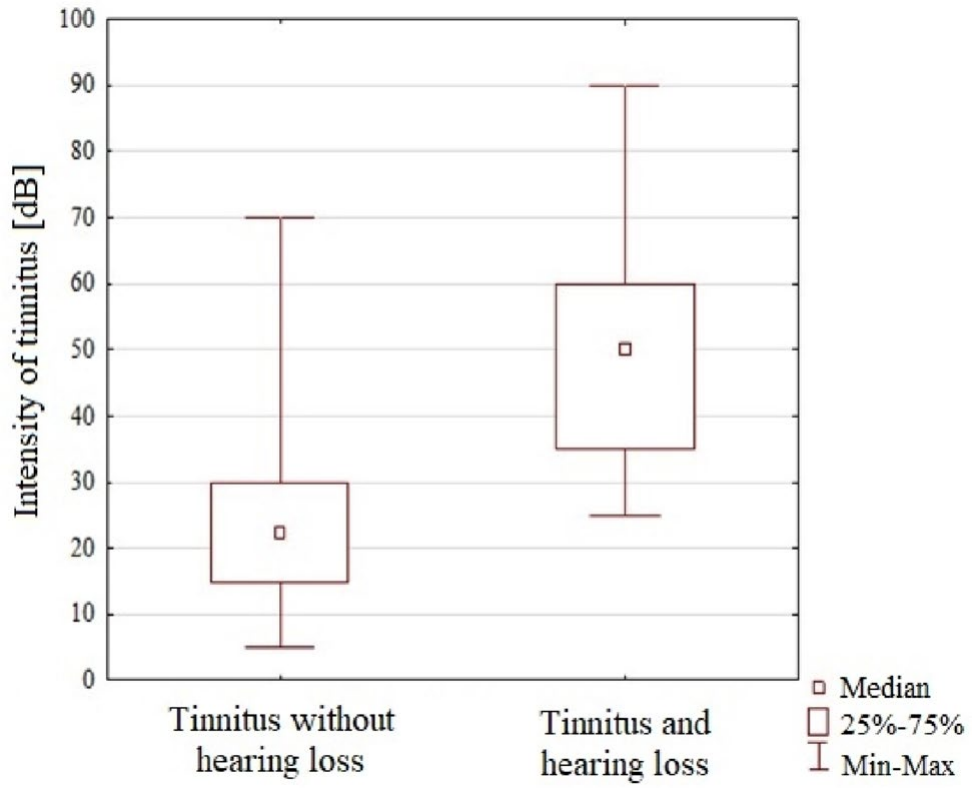
The graphical distribution of tinnitus intensity in the examined patients with the division into study groups

A graphical presentation of the results is given in Fig. 4, and their analysis is included in Table 4.

**Table 4:** The comparative analysis of tinnitus frequency and intensity between the examined groups

Parameter	Test result	*P* value
Intensity of tinnitus	− 6.152	< 0.001
Frequency of tinnitus	0.241	0.809

## Discussion

There are no reports in the literature on the effect of factors such as gender on the presence of tinnitus or hearing loss [10–14]. Our research confirmed the relationship between age and the incidence of tinnitus with hearing loss, and tinnitus without hearing loss. The study showed statistically significant differences in the age distribution from the normal distribution for the control group, and patients with tinnitus and hearing loss. The median age was 34 years for the control group, 39 years for the group with tinnitus and normal hearing, and for the group with tinnitus and hearing loss—55 years. The last group analyzed was the oldest, with patients ranging in age from 26 to 73 years.

It is interesting to note that the results revealed a statistically significant difference between the control group as well as for patients with tinnitus and hearing loss, as shown in Table 2. Taking these data into account, it can be concluded that with age, hearing loss is more common and the chance of developing tinnitus increases. A similar relationship was observed when comparing the group with tinnitus and normal hearing to the group with tinnitus and hearing loss. It is most likely, therefore, that it is not age alone that is the etiological factor for hearing loss and tinnitus, but also comorbidities that can directly affect hearing and thus cause hearing deprivation.

However, there was no relationship between age and the control group, and the group with tinnitus and normal hearing. Further analysis showed that hearing impairment and the onset of tinnitus was closely related to abnormal results of HFA, i.e., in the range of 8–16 kHz. Therefore, tinnitus can be treated as the first symptom of the onset of hearing high-frequency hearing loss, which is confirmed by the literature [13, 15, 16]. This, in turn, leads to the conclusion that HFA should be included in the audiological diagnostic panel, especially for patients with tinnitus.

Taking into account the characteristics of tinnitus, i.e., its intensity and frequency, we made a comparison of their parameters. This analysis was performed in the groups of patients with tinnitus.

The analysis of the frequency parameters did not show significant differences. The mean values for the group without hearing loss were slightly higher at 3.95 kHz, with a median at 3 kHz. The results obtained for patients with tinnitus and hearing loss were 2.94 kHz for the mean and 2 kHz for the median.

The results obtained in the examined groups showed that hearing loss did not have a significant effect on the frequency of tinnitus. However, it turned out that the occurrence of hearing loss in the frequency range up to 8 kHz significantly affected tinnitus intensity—its volume.

The results achieved for tinnitus intensity showed a significant difference for the mean and median values in the compared groups. Patients with tinnitus alone achieved a mean of 24.16 dB and a median of 22.5 dB. In the case of patients with tinnitus and hearing loss, the mean and median values were twice higher, reaching to 50.00 dB.

Considering the fact, that the volume of tinnitus depends on the frequency range of hearing loss in PTA, HFA becomes a very valuable diagnostic tool, especially in assessing the possibility of predicting the onset of tinnitus. The world literature indicates that in the case of patients with hearing loss observed in a wide frequency range, tinnitus is more bothersome, most likely due to its louder nature [17–20]. It is therefore all the more important to include HFA in the scope of diagnostic tests in tinnitus patients to enable diagnosis, treatment and, above all, prevention of tinnitus.

## Conclusion

Tinnitus may be a symptom that indicates the occurrence of high-frequency hearing loss due to the fact that the frequency range of hearing loss widens with age, so HFA should be performed as a routine audiological test in patients with tinnitus.

## Data Availability

All data used to support the finding of this study are available from the corresponding author upon request.
